# Assessing the transition from intravenous to subcutaneous delivery of rituximab: Benefits for payers, health care professionals, and patients with lymphoma

**DOI:** 10.1371/journal.pone.0261336

**Published:** 2022-01-24

**Authors:** Michael J. Harvey, Yi Zhong, Eric Morris, Jacob N. Beverage, Robert S. Epstein, Anita J. Chawla

**Affiliations:** 1 Analysis Group, Inc., London, United Kingdom; 2 Analysis Group, Inc., Menlo Park, California, United States of America; 3 Halozyme Therapeutics, San Diego, California, United States of America; 4 Epstein Health, LLC, Woodcliff Lake, New Jersey, United States of America; Istanbul University-Cerrahpaşa, Cerrahpaşa Faculty of Medicine, TURKEY

## Abstract

Subcutaneous (SC) administration of rituximab provides an opportunity for reduced patient treatment burden and increased healthcare efficiencies as an alternative to intravenous (IV) rituximab. There is minimal evidence comparing costs associated with SC and IV rituximab in a US setting. This research assessed the impact of transitioning patients from IV to SC rituximab for treatment of non-Hodgkin’s lymphoma (NHL) from the US payer, provider, and patient perspective. We developed a model to estimate cost differences for transitioning 20% of a patient cohort from IV to SC rituximab. We included patients with incident diffuse large B-cell lymphoma, incident and recurrent follicular lymphoma, and incident and recurrent chronic lymphocytic leukemia. In the model, each patient received the same number of doses and that there was no difference in discontinuation between cohorts due to non-inferior efficacy and a similar safety profile. Model inputs were collected from published literature and publicly available data. Scenario analyses tested the impact of availability of low-cost biosimilars. In the base case (1,000,000 covered lives), we estimated a total of 157 patients, with 769 total drug administrations. A transition of 20% of patients from IV to SC was projected to generate $153,000 in payer savings, increase provider capacity by 270 hours, and free 470 hours of patient time. Scenario analyses suggest SC administration will be cost saving for payers even with a market where biosimilars approach 50% market share. A 20% transition to SC rituximab in a single cohort of patients has the potential to generate significant US health system value in the form of payer savings, increased practice capacity, and patient time.

## Introduction

Subcutaneous (SC) administration of therapeutic proteins is an established route of administration for an array of therapeutic areas and for multiple indications [1]. Historically, intravenous (IV) infusion has afforded rapid and accurate administration of drug into the systemic circulation without the delay associated with the absorption process [2]. Nevertheless, SC administration has potential advantages over IV administration including reduced administration time, decreased patient discomfort, reduced drug wastage, and potentially fewer medication errors due to fixed dosing [2–7]. In addition, SC administration offers the possibility for treatment outside of a clinic setting [8].

In the US, SC formulations of monoclonal antibodies (mAbs) have been approved for a variety of indications including the treatment of oncologic and rheumatologic conditions [1]. Recombinant human hyaluronidase PH20 (rHuPH20) is an enzyme that transiently degrades the hyaluronan (HA) in the SC space around an injection site, enabling the administration of large volumes with a single SC injection [9]. This approach allows for the rapid subcutaneous delivery of larger volume injections of biologics and small-molecule drugs, providing the potential to decrease the burden of treatment for patients that IV administration may pose while delivering comparable efficacy and safety.

The monoclonal antibody rituximab, first approved in 1997, is a standard of care for a number of lymphoma and leukemia indications [10, 11]. In 2017, the US Food and Drug Administration (FDA) approved the combination of SC rituximab (rituximab plus rHuPH20) for adult patients with follicular lymphoma (FL), diffuse large B-cell lymphoma (DLBCL), and chronic lymphocytic leukemia (CLL) [12]. IV infusion times typically range from 1.5–4 hours but can be as long as 6 hours depending on the indication, dosing, and infusion history [13, 14]. SC rituximab provides patients an option that considerably shortens administration times to approximately 5–7 minutes [12]. Time and motion studies conducted in 8 countries showed a 74% average reduction in patient time in the clinic for SC compared to IV rituximab [13]. Results from patient surveys showed more than 80% of patients preferred SC after experiencing both SC and IV formulations [7]. In addition to patient benefits, SC rituximab uses indication-specific flat dosing (1400mg for DLBCL and FL, or 1600mg for CLL) which can save the provider preparation time, as well as costs by eliminating wastage [11, 12].

The potential economic impact of SC rituximab to reduce treatment burden and administration costs is less well documented in US settings [15, 16] compared with ex-US settings [4, 13, 17–26]. The objective of this research was to assess transitioning from IV to SC administration of rituximab for treatment of DLBCL, FL, and CLL from the US payer, provider, and patient perspectives.

## Methods

To estimate the incremental cost differences between IV and SC rituximab in a simulated cohort of non-Hodgkin’s lymphoma (NHL) patients with incident DLBCL, FL, CLL, or recurrent FL or CLL over the course of 6–8 cycles of therapy (depending on indication), we developed a decision-analytic cost model. The cost model was built using Microsoft Excel 2016 (Microsoft, Redmond, WA). In the base case, the model was used to evaluate two distinct scenarios. In Scenario 1, we assumed 100% of the cohort was treated with IV rituximab. In Scenario 2, we assumed 20% of the patient cohort was transitioned to SC rituximab, and the remaining 80% were treated with IV rituximab. We assumed that each patient received the same number of doses; there was no difference in discontinuation between the IV and SC cohort. Clinical trial data showed non-inferior efficacy and a similar safety profile between the IV and SC rituximab [27–29]; we excluded adverse event (AE) rates and their respective costs from our analyses assuming equivalence in both IV and SC groups. Because the model only included a single cohort over a short time horizon, discounting was excluded.

All model calculations were based on the patient pathway for rituximab treatment across the three stages of service delivery: pre-service, intra-service, and post-service. The model was constructed from the perspective of payers (including both Medicare and commercial payers), health care providers (assuming administrations are office-based with a fee-for-service [FFS] payment model), and the patients. Primary model analyses were conducted at the administration level. Secondary analyses were conducted at the cohort level where primary analyses were scaled by the expected number of administrations per patient and the number of patients. The model outcomes of interest were total direct medical care costs (payer perspective), expected cost savings (payer perspective), and expected staff and patient time savings (provider and patient perspectives, respectively).

### Patient population

All model inputs are presented in Table 1. The patient cohort in the base case was defined as the estimated number of lymphoma patients in a hypothetical health plan with 1,000,000 covered lives. Based on the labelled indication for SC rituximab, the cohort consisted of NHL patients with incident DLBCL, FL, or CLL, and recurrent FL or CLL [12]. Surveillance, Epidemiology, and End Results Program (SEER) data were used to estimate the patient cohort, with adjustments applied by an age and sex distribution based on the US Census Bureau’s national population projection data from 2018.

**Table 1:** Model inputs.

Parameter	Value		Reference
***Health plan characteristics***			
Patient population in health care plan, n	1,000,000		Assumption
Men < 65 years, %	39.6		US Census Bureau [30]
Men ≥ 65 years, %	9.6		US Census Bureau [30]
Women < 65 years, %	38.6		US Census Bureau [30]
Women ≥ 65 years, %	12.2		US Census Bureau [30]
Payer mix, (Medicare:commercial), %	21.8:78.2		Assumption based on age distribution
Patient body surface, m^2^	1.86		Baker [31]
***Market share***			
Rituximab IV–Branded, %	80		Amgen Inc. [32]
Rituximab IV–Biosimilar 1, %	17		Amgen Inc. [32]
Rituximab IV–Biosimilar 2, %	3		Amgen Inc. [32]
***Epidemiology***			
NHL annual incidence (per 100,000)			
Men < 65 years	12.19		US Census Bureau [30]
			Howlader [33]
Men ≥ 65 years	106.40		US Census Bureau [30]
			Howlader [33]
Women < 65 years	9.22		US Census Bureau [30]
			Howlader [33]
Women ≥ 65 years	72.73		US Census Bureau [30]
			Howlader [33]
NHL cases with DLBCL, %	22.92		Howlader [33]
NHL cases with FL, %	11.46		Howlader [33]
Chronic lymphocytic leukemia, %	19.73		Howlader [33]
***Time and motion data (patient perspective)*** ^***a***^			
Pre-service, minutes	IV	SC	
Review patient chart and notes	2.9	2.9	Young [34]
Patient scheduling and secretary time	13.2	13.2	Shinder [35]
Vial and consumable collection	2	2.2	De Cock [13]
Therapy preparation - Reconstitution of IV Rituximab or preparation of Rituximab syringe	12.5	8.6	De Cock [13]
Intra-service (therapy administration), minutes	IV	SC	
Install peripheral access/line flushing	6.6	0	De Cock [13]
Pre-medication administration	8.3	2.5	De Cock [13]
Infusion initiation	3.75	0	De Cock [13]
Chair time (infusion/injection time)^b^	172.13	5	Rituxan Prescribing Information [36]
			Rituxan Hycela Prescribing Information [12]
Infusion monitoring (staff interaction rate)	64%		Pierce [37]
Disconnect and flush IV line/dispose materials	3.6	0	De Cock [13]
Post-service			
Post-infusion/injection monitoring	1.65	15	Rituxan Hycela Prescribing Information [12]
Discharge, complete notes, and charting	11	11	Shinder [35]
***Costs*, *$***			
DLBCL + FL drug cost, cost per dose	ASP		
Rituximab SC	5,632		Centers for Medicare & Medicaid Services [38]
Rituximab IV—Branded	6,388		Centers for Medicare & Medicaid Services [38]
Rituximab IV–Biosimilar 1	4,795		Centers for Medicare & Medicaid Services [38]
Rituximab IV–Biosimilar 2	4,713		Centers for Medicare & Medicaid Services [38]
CLL drug cost, cost per dose	ASP		
Rituximab SC	6,437		Centers for Medicare & Medicaid Services [38]
Rituximab IV	9,126		Centers for Medicare & Medicaid Services [38]
Rituximab IV–Biosimilar 1	6,850		Centers for Medicare & Medicaid Services [38]
Rituximab IV–Biosimilar 2	6,732		Centers for Medicare & Medicaid Services [38]
Drug administration, unit cost per dose ($)			
Rituximab SC, Medicare^c^	82.35		Centers for Medicare & Medicaid Services [39]
Rituximab SC, Commercial	86		Hansen [15]
Rituximab IV 1 hour, Medicare^d^	148.30		Centers for Medicare & Medicaid Services [39]
Rituximab IV additional hour, Medicare^d^	31.40		Centers for Medicare & Medicaid Services [39]
Rituximab IV, DLBCL + FL, Commercial	361		Hansen [15]
Rituximab IV, CLL, Commercial	418		Hansen [15]
Consumables, total cost (IV/SC)	15.62	5.87	Micromedex Red Book [40]
			MedEx Supply [41]
			Mountainside Medical Equipment [42]
			USA Medical and Surgical Supplies [43]
			Westend Medical [44]

Abbreviations: CLL, chronic lymphocytic leukemia; FL, follicular lymphoma; DLBCL, diffuse large B-cell lymphoma; NHL, non-Hodgkin’s lymphoma; IV, intravenous therapy; SC, subcutaneous injection; US, United States; WAC, wholesale acquisition cost.

^a^ Time and motion data was only used to calculate administration costs in the provider and policymaker perspectives. Payer perspective drug administration costs can be found in the costs section of Table 1.

^b^ Patient infusion chair time was calculated based on the amount of drug required for a patient with a body surface of 1.8 m^2^ and a standard infusion rate of 100 mg/hr. In the absence of infusion toxicity, increase rate by 100 mg/hr increments at 30-minute intervals, to a maximum of 400 mg/hr. 5% of patients were assumed to undergo rapid infusion in subsequent cycles, where 20% of the total dose is administered over the first 30 min (75 mg/m^2^) and then 80% of total dose over following 60 min (300 mg/m^2^).

^c^ SC administration cost was assumed to be the cost of chemotherapy administration, subcutaneous or intramuscular; non-hormonal anti-neoplastic (HCPCS code: 96401).

^d^ IV administration cost was assumed to be chemotherapy administration, intravenous infusion technique; up to 1 hour, single or initial substance/drug (HCPCS code: 96413) plus chemotherapy administration, intravenous infusion technique; each additional hour (HCPCS code: 96415).

### Drug administration

Administration of rituximab included initial administrations and subsequent administrations. Initial administrations were defined as the first dose of rituximab for patients initiating treatment. Subsequent administrations were defined as any administration of rituximab after the initial administration. Based on the product label for rituximab, all initial administrations in Scenarios 1 and 2 were assumed to be IV. We assumed incident DLBCL, incident FL, and both incident and recurrent CLL patients receive 6 cycles of therapy on average, and recurrent FL patients receive 8 cycles of therapy on average [15]. Both branded and biosimilar products were included in the analysis. When scaled to the cohort level, the total number of administrations was adjusted to account for market share of rituximab within the specific indication.

### Costs

Costs associated with the administration of rituximab were included in the model, including costs for drug therapy and administration. Costs for co-administered products were excluded, and no conclusions or inferences were made about co-administered products. All costs in the model were specified in 2021 USD. From the payer perspective, average sales price (ASP) for drug therapies was sourced from the Centers for Medicare and Medicaid Services’ (CMS) ASP January 2021 data files [40]. In the base case, we assumed drug therapies were reimbursed at 4.3% above ASP in the Medicare population, and at 8.6% above ASP in the commercial population [45]. Administration costs were based on reimbursement, using the health care common procedure coding system (HCPCS) for the year 2021 [15]. Reimbursement for Medicare patients was sourced from the CMS Physician Fee Schedule and commercial reimbursement rates were sourced from the available published literature [15]. From the health care provider perspective, a micro-costing approach was used based on administration times sourced from the published literature. This micro-costing approach accounted for the time (in minutes) spent on different activities across the stages of service delivery (pre-service, intra-service, post-service), as well as the per minute cost of the staff member who performed the activity. The time data were combined with hourly wage data from the Bureau of Labor Statistics (BLS) to generate the costs of administration across the stages of service delivery [46]. Costs for consumables and other medical supplies needed for rituximab administration were sourced from publicly available medical supply price lists and Redbook [40–44, 47]. Where possible, costs for multipack items were used to reduce the overall consumables costs. For the patient perspective, the same time and motion analysis was used but timings were adjusted to account for patient rather than staff time. This approach accounted for the time (in minutes). Results for expected time in clinic were reported from the patient perspective at the per administration and cohort level.

### Sensitivity analyses

We conducted three sensitivity analyses within the model. First, we varied the share of patients transitioned to SC rituximab from IV (from 10% to 50%) with the objective of assessing the total amount of time that could be saved from the provider and the patient perspectives, respectively. Second, we tested the impact of biosimilar adoption in the current market. In this analysis, we simultaneously varied the likelihood of transitioning to SC (from 0% to 50%) and from a branded IV product to a biosimilar IV product (from 0% to 50%). The objective of this analysis was to determine the effect of biosimilar uptake on the potential cost savings associated with transitioning patients to branded SC, for the provider and the payer. Finally, we adjusted the percentage of patients receiving rapid infusion for DLBCL and FL to 50% and 100%, respectively. The objective of this analysis was to understand both the implications for cost savings and time savings if more patients were transitioned to rapid infusion.

### Disaggregated and scaled analyses

There are multiple therapeutics approved for the treatment of DLBCL, FL, and CLL. In the base case and in the sensitivity analyses, results are presented at the cohort level; however, because rituximab may not be used to treat all patients given the available options, we included a set of disaggregated (DLBCL+FL, CLL) and scaled (the per administration level) results. For this set of analyses, the sensitivity analyses at the disaggregated and scaled level were reproduced.

## Results

Model results are presented in Table 2. In the base case, there were an estimated 157 NHL patients in a plan of 1,000,000 individuals (incident: 63 DLBCL, 31 FL, 54 CLL; recurrent: 6 FL, 3 CLL). Among these patients, the expected number of total drug administrations was 769 (350 DLBCL, 235 FL, 184 CLL). From the payer perspective, a transition of 20% of patients from IV to SC was projected to save $153,000 in total. Within projected savings, $71,000 was attributable to DLBCL and FL patients and $82,000 was attributable to CLL patients. From the provider perspective, the transition from IV to SC was also projected to be cost saving for the practice ($124,000) and to increase provider capacity by 270 hours of staff time (195 DLBCL + FL, 75 CLL). From the patient perspective, an SC administration is estimated to have a total duration of 60 minutes across all levels of services, regardless of indication. For DLBCL and FL, an IV infusion is estimated to have a duration of 234 minutes; for CLL, an IV infusion is estimated to have a duration of 273 minutes. At the cohort level, a 20% transition from IV to SC was projected to save approximately 470 hours of patient time.

**Table 2:** Base-case results.

Costs–Payer	Scenario 1 ($)	Scenario 2 ($)	Difference ($, Scenario 2–1)
Total	5,803,000	5,650,000	-153,000
DLBCL + FL	4,020,000	3,949,000	-71,000
CLL	1,783,000	1,701,000	-82,000
Costs–Provider			
Total	5,248,000	5,124,000	-124,000
DLBCL + FL	3,630,000	3,578,000	-52,000
CLL	1,618,000	1,546,000	-72,000

Abbreviations: CLL, chronic lymphocytic leukemia; FL, follicular lymphoma; DLBCL, diffuse large B-cell lymphoma.

Results for DLBCL and FL are grouped because dosing for these products is equivalent for both IV and SC products.

### Sensitivity analyses

**Fig 1:**
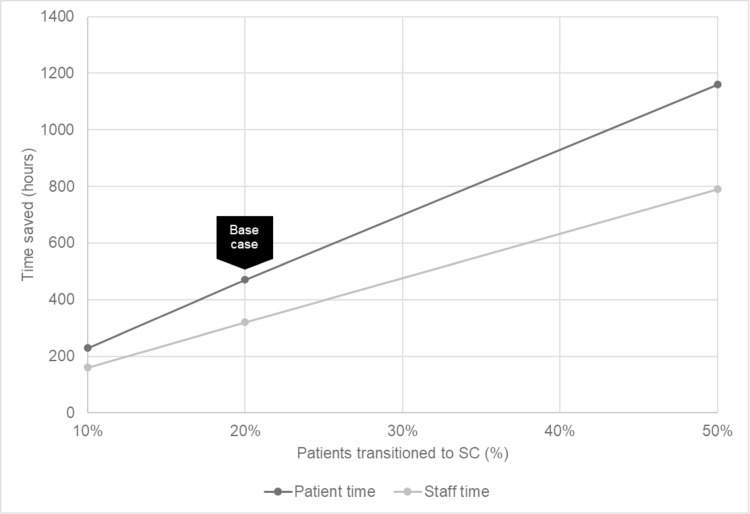
Expected time savings, all patients.

**Fig 2:**
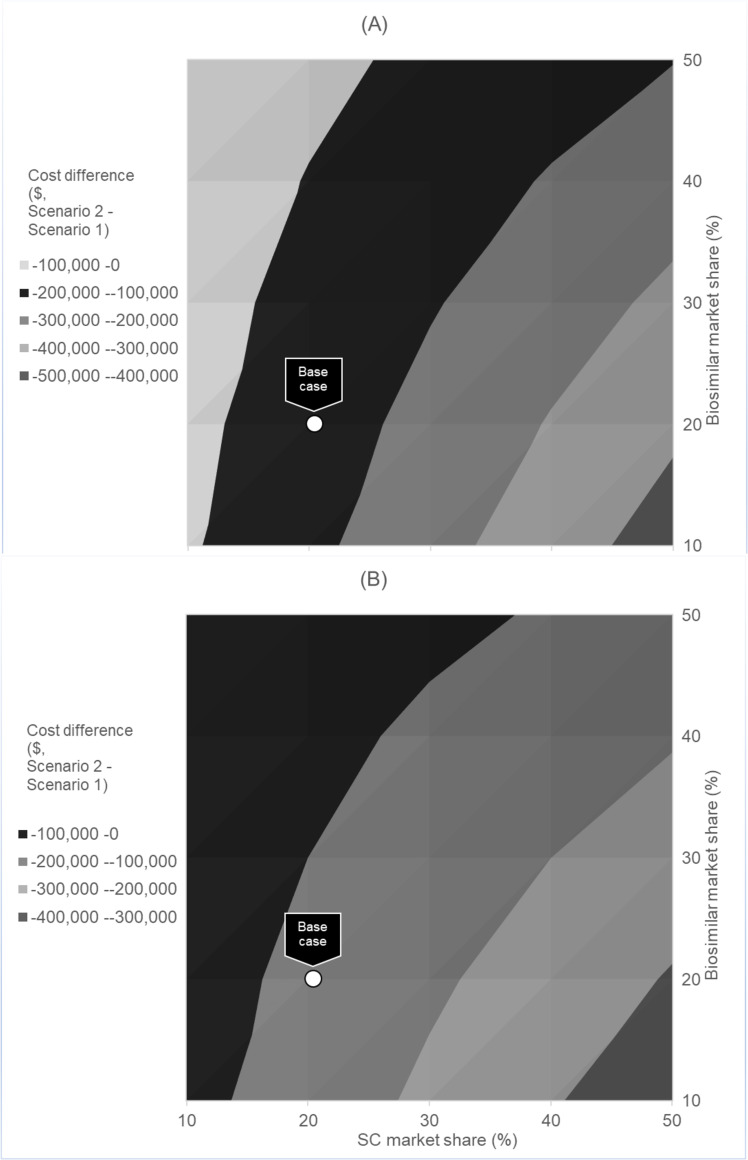
Expected cost savings given market share shift in SC and biosimilar IV, all patients.

**Fig 3:**
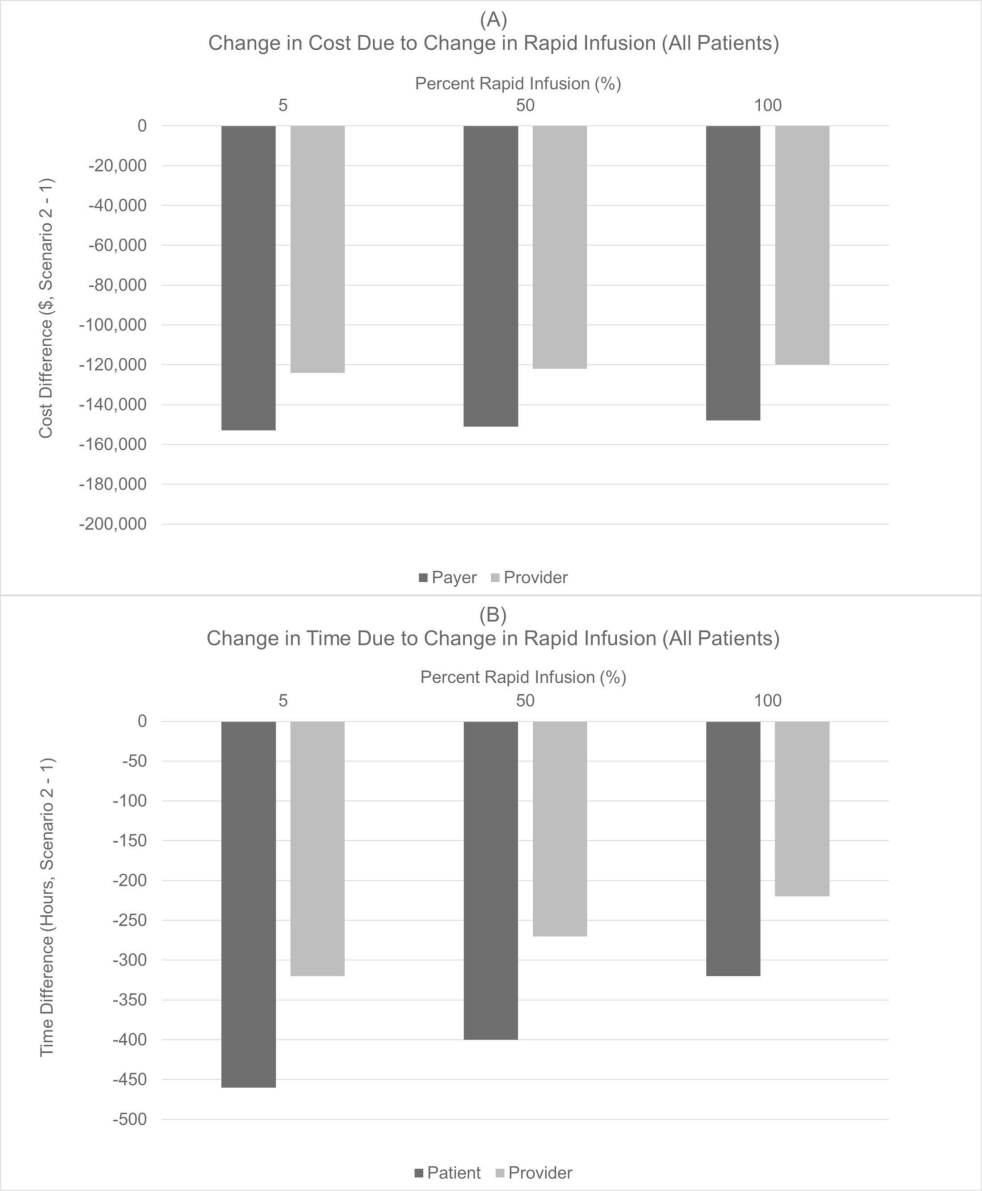
Expected cost and times savings given increased use of rapid infusion, all patients.

Results from the sensitivity analyses are shown in Figs 1–3. Assuming 10% of patients transition to SC from IV, savings of 160 hours of staff time and 230 hours of patient time would be expected—approximately 4 weeks and 6 weeks of full-time equivalent work, respectively (Fig 1). If 50% of patients were transitioned to SC from IV, a total of 790 hours of staff time and 1160 hours of patient time could be saved.

Results from the multidimensional sensitivity analysis on market share for SC compared to biosimilar IV are presented in Fig 2. At the cohort level, across all ranges of market share evaluated, transitioning patients to SC rituximab will always be cost saving to the payer (Fig 2, Panel A). The greatest cost savings can be achieved when the proportion of biosimilars in the market is relatively low and the transition to SC is relatively high. Panel B in Fig 2 shows results from the provider perspective. Similar to the payer perspective, across all ranges of market share evaluated, transitioning patients to SC rituximab is estimated to always be cost saving to the provider.

A payer can be expected to save $151,000 with a 20% transition of patients to SC administration with 50% of the IV patients receiving rapid infusion (Fig 3). Under these conditions, the expected savings associated with transitioning from IV to SC are $2,000 lower. Even under the assumption of 100% of IV patients receiving rapid infusion rituximab, a 20% transition of patients to SC results in cost and time savings for the payers, providers, and patients.

### Disaggregated and scaled analyses

**Fig 4:**
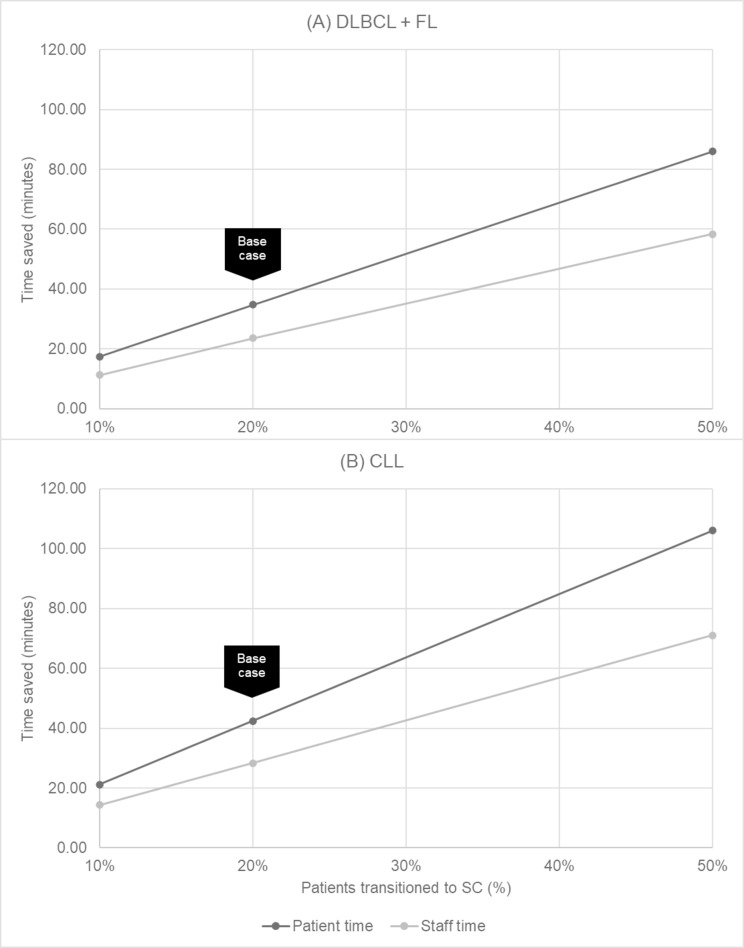
Expected time savings, per indication, per administration.

**Fig 5:**
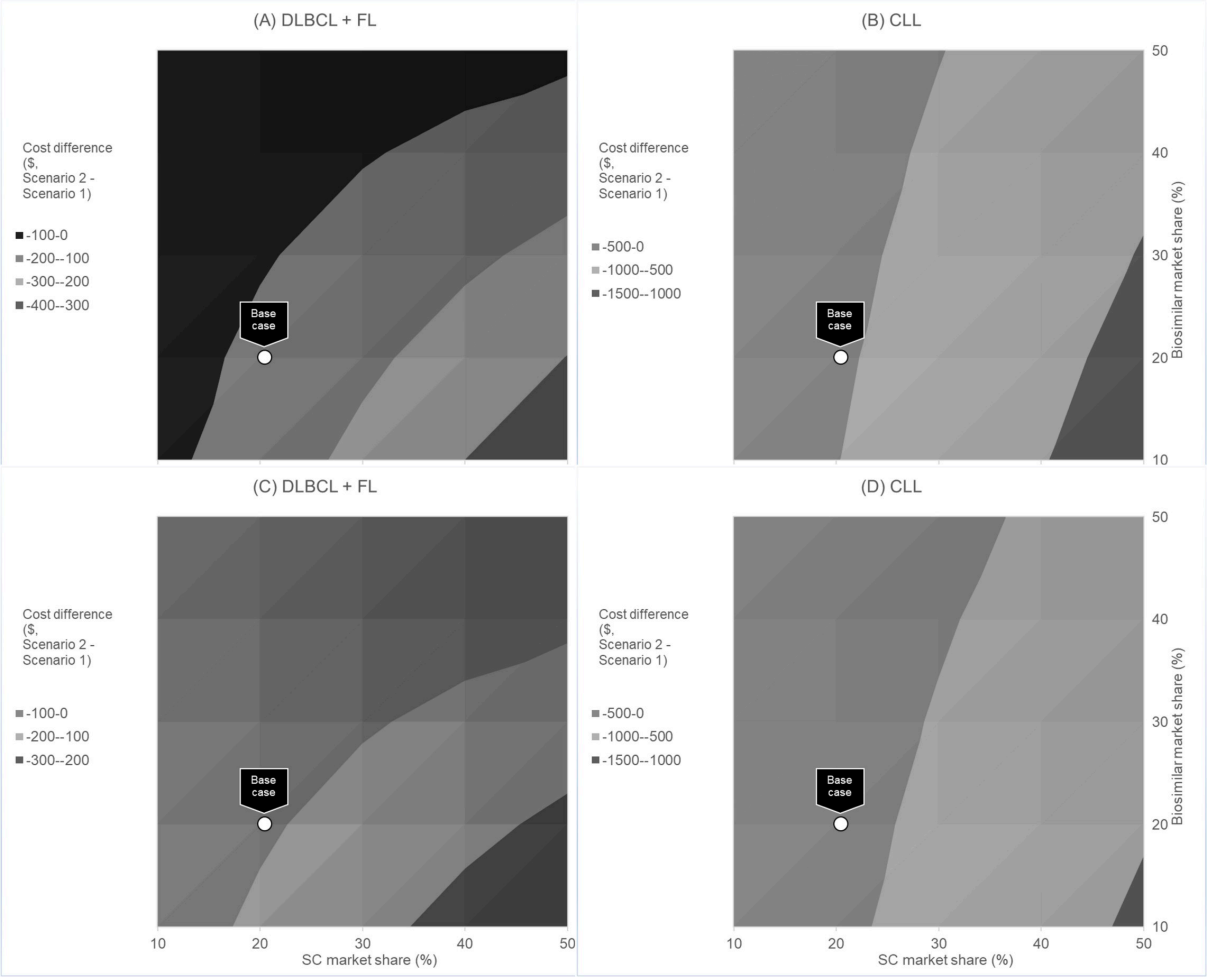
Expected cost savings given market share shift in SC and biosimilar IV, per indication, per administration.

At the per administration level, varying the percentage of patients transitioning to SC between 10% and 50% saved between 17 minutes and 58 minutes for DLBCL+FL patients, and saved between 21 minutes and 108 minutes for CLL patients, respectively (Fig 4). When considering the impact of biosimilar uptake on the market, in all scenarios tested, adding SC to the treatment mix was cost saving to payers (Fig 5, Panels A and B) and providers (Fig 5, Panels C and D). Cost savings were greater at the per administration level for CLL compared with DLBCL+FL. Under the assumption of 100% of DLBCL+FL patients receiving IV rituximab, a 20% transition of patients to SC administration would result in savings of $113 per administration, and $82 per administration for payers and providers, respectively. In addition, patients would be expected to save 18 minutes per administration, and providers would expect to save 13 minutes per administration. The prescribing information for rituximab does not include an indication for rapid infusion in the treatment of CLL; therefore, cost and time savings given a shift in rapid infusion were only reported for DLBCL+FL patients.

## Discussion

The objective of this research was to estimate the payer costs, provider costs, and the potential time savings that could be realized by transitioning patients from IV to SC rituximab. From a payer perspective, the base case—20% transition to SC rituximab in a single cohort of NHL patients—has the potential to save more than $150,000 over the treatment course for the cohort. These potential savings to the system are attributable to lower ASP in the base case as well as shorter administration time in a health care setting [4].

We identified two US-based studies comparing SC and IV rituximab, both presenting results from the payer perspective only [15, 16]. In the first study with a three-year horizon, SC rituximab lowered payer costs by $230,000 in a cohort of 150 patients when SC rituximab had approximately 50% market share [15]. In our model, we estimated approximately $150,000 saved in a cohort of 157 patients with a 20% transition from IV to SC rituximab. Based on the results from the sensitivity analysis (Fig 2, Panel A) at 50% SC market share would result in $300,000 to $500,000 of savings to the payer, if biosimilar market share is less than 30%. In the second study, time savings of 130 minutes per cycle of treatment were reported [16]. Our model generated similar results with 120 minutes saved per DLBCL + FL administration, and 140 minutes per CLL administration.

The impact of SC administration on service delivery and the downstream impact on patients and patient experience is particularly important to consider in care of patients with cancer. Given the potential for cancer patients to be more sensitive to overall service performance, continuous improvement of the service delivery should be seen as an imperative, especially considering that poor service experiences can negatively amplify the already difficult experience of an oncology patient [48]. The model highlights the potential impact on service delivery with a conservative 20% transition to SC, saving 470 hours of patient time (more than 11 weeks of full-time equivalent work). The time saved is realized regardless of the potential share that biosimilars are expected to accrue since all biosimilars are administered intravenously. While this model highlights the potential for SC rituximab to save costs from a payer perspective while providing non-inferior efficacy to the patient thus providing an innovative option for care delivery [3, 13, 27–29], time saved is only one element of patient experience. Factors such as injection pain and treatment adherence should be considered and quantify when assessing patient experience [49].

**Fig 6:**
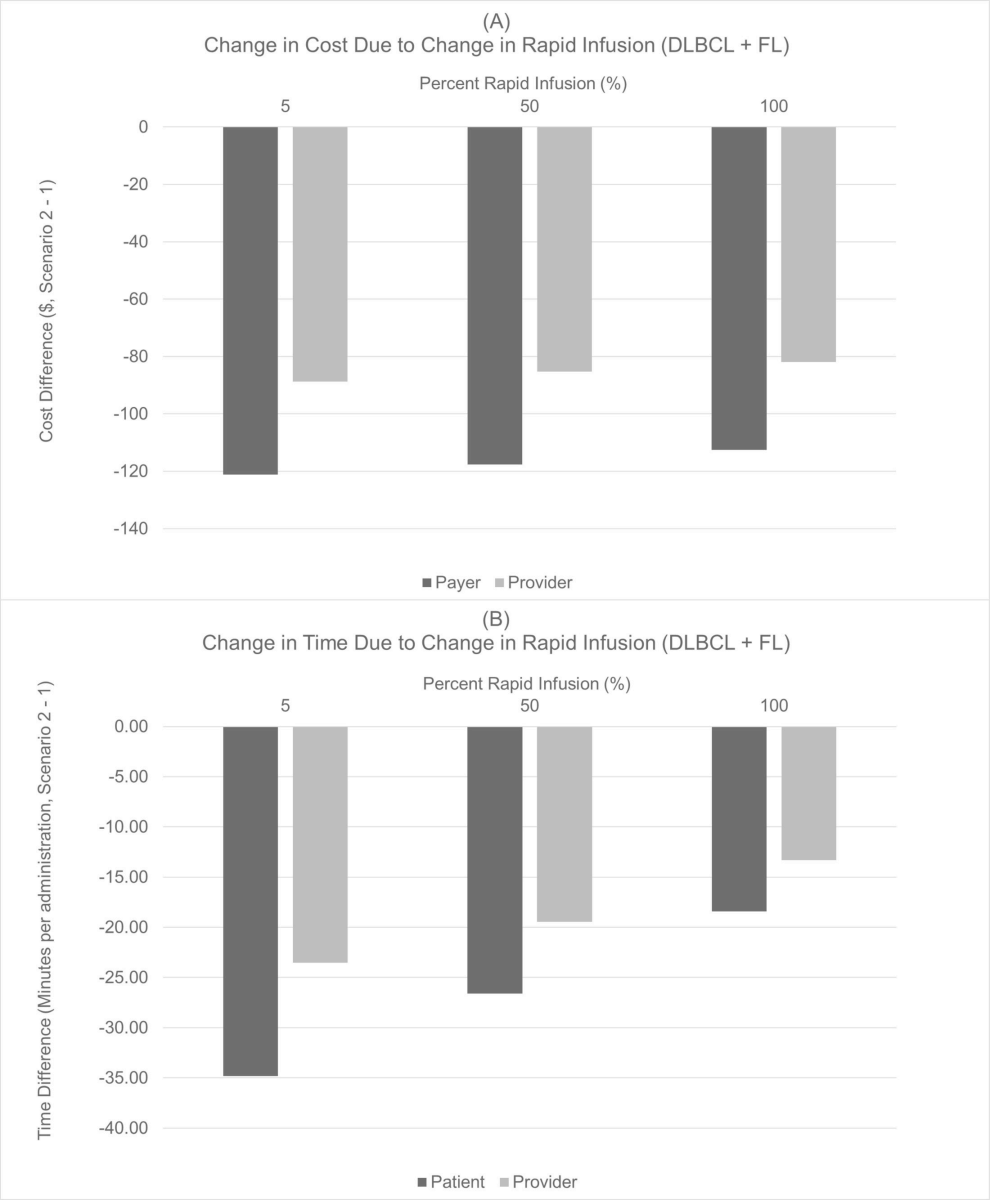
Expected cost and times savings given increased use of rapid infusion, per indication, per administration.

Our findings are dependent on specific assumptions that informed our approach and may not be generalizable to all settings. First, model results in this analysis do not account for variations in body surface area (BSA). In the model, we used an average BSA, but because IV rituximab dosing is BSA-based, it is possible some payers, based on the BSA of their patient population could generate more or less savings than the estimates provided in this work. The fixed dosing of SC rituximab can be a benefit from a planning perspective; fixed dosing removes a source of variation (BSA), which makes estimating potential costs more straightforward. For example, CLL patients contributed fewer overall patients (57 CLL patients / 157 total patients), and fewer administrations (184 administrations for CLL patients / 769 administrations in total), but the cost savings for CLL patients was still greater compared with DLBCL and FL patients. This result was due to the cost of therapy; the volume of IV rituximab required for the modelled CLL patients raised the cost such that the fixed dose SC rituximab was less costly than both the branded and biosimilar IV rituximab in the base case. Second, we presented results at a cohort level, but it is possible that other treatments beyond rituximab could be used to treat DLBCL, FL, and CLL patients. For transparency, we included disease-specific results in Table 1, and a full set of disaggregated and scaled results in Figs 4‒6. In all situations, we found that adding SC to the treatment mix would be time saving for patients and providers, and cost saving for payers and providers. Third, we only considered FFS payment models in the analysis. We used publicly available FFS payment data in the model; therefore, the results may not be generalizable to payment models other than those that are FFS. Fourth, we did not consider the specific clinical setting in the model. IV treatments are most likely completed in an infusion suite, but it is unlikely that an infusion suite would be needed to administer SC rituximab. Factoring in capital costs for an infusion suite versus a standard outpatient clinic room into the analysis, for example, is an area for future research. Finally, we did not model non-clinical settings. We assume all administrations were conducted in a clinical setting. Given the increasing importance of telemedicine and home-based administrations, especially in the era of the SARS-CoV-19 pandemic, adding a perspective outside the clinical setting could highlight additional value not captured in this analysis.

## Conclusion

The model serves as a framework for further analyses of new therapeutics with SC administration as they are introduced. Notably, the FDA recently approved daratumumab and hyaluronidase–fihj for the treatment of multiple myeloma and pertuzumab, trastuzumab, and hyaluronidase–zzxf for the treatment of breast cancer [50, 51]. SC administration is expected to reduce caregiver and patient burden significantly and to contribute value to the health care system overall. As the US health care system continues to grow and leverage new technology, SC delivery of therapeutics is expected to expand with great potential to improve patient experience, lower the cost of care, and help optimize the delivery of care [52].

## Supporting information

S1 File (PDF)
