# Supplementary material for: Assessing the transition from intravenous to subcutaneous delivery of rituximab: Benefits for payers, health care professionals, and patients with lymphoma
Source: PLoS One. 2022 Jan 24;17(1):e0261336. doi: 10.1371/journal.pone.0261336 (PMC8786206; doi:10.1371/journal.pone.0261336)
Supplement: S1 File — (PDF) [file pone.0261336.s001.pdf]

Table 2

Costs – Payer	Scenario 1 ($)	Scenario 2 ($)	Difference ($, Scenario 2 - 1)
Total	5,803,000	5,650,000	-153,000
DLBCL + FL	4,020,000	3,949,000	-71,000
CLL	1,783,000	1,701,000	-83,000
Costs – Provider			
Total	5,248,000	5,124,000	-124,000
DLBCL + FL	3,630,000	3,578,000	-52,000
CLL	1,618,000	1,546,000	-72,000

Figure 1

Percent of patients transitioned to SC (%)	Patient Time (hours)	Staff Time (hours)
10%	230	160
20%	470	320
50%	1,160	790

Figure 2 **Panel A - Payer Perspective**

CLL, Difference ($, Scenario 2 - 1)	SC Market Share (%)				
BS Market Share (%)	10%	20%	30%	40%	50%
10%	-45,043	-90,086	-135,128	-180,171	-225,214
20%	-41,263	-82,525	-123,788	-165,051	-206,313
30%	-37,483	-74,965	-112,448	-149,930	-187,413
40%	-33,702	-67,405	-101,107	-134,810	-168,512
50%	-29,922	-59,845	-89,767	-119,689	-149,612

DLBCL & FL, Difference ($, Scenario 2 - 1)	SC Market Share (%)				
BS Market Share (%)	10%	20%	30%	40%	50%
10%	-43,966	-87,932	-131,897	-175,863	-219,829
20%	-35,364	-70,729	-106,093	-141,458	-176,822
30%	-26,763	-53,526	-80,289	-107,052	-133,815
40%	-18,162	-36,323	-54,485	-72,646	-90,808
50%	-9,560	-19,120	-28,681	-38,241	-47,801

Combined, Difference ($, Scenario 2 - 1)	SC Market Share (%)				
BS Market Share (%)	10%	20%	30%	40%	50%
10%	-89,009	-178,017	-267,026	-356,034	-445,043
20%	-76,627	-153,254	-229,881	-306,508	-383,135
30%	-64,246	-128,491	-192,737	-256,982	-321,228
40%	-51,864	-103,728	-155,592	-207,456	-259,320
50%	-39,483	-78,965	-118,448	-157,930	-197,413

Figure 2 **Panel B - Provider Perspective**

CLL, Difference ($, Scenario 2 - 1)	SC Market Share (%)				
BS Market Share (%)	10%	20%	30%	40%	50%
10%	-39,145	-78,290	-117,435	-156,580	-195,725
20%	-35,634	-71,268	-106,902	-142,536	-178,170
30%	-32,123	-64,246	-96,369	-128,492	-160,615
40%	-28,612	-57,224	-85,836	-114,448	-143,061
50%	-25,101	-50,202	-75,303	-100,404	-125,506

DLBCL & FL, Difference ($, Scenario 2 - 1)	SC Market Share (%)				
BS Market Share (%)	10%	20%	30%	40%	50%
10%	-33,822	-67,643	-101,465	-135,287	-169,109
20%	-25,833	-51,665	-77,498	-103,331	-129,164
30%	-17,844	-35,688	-53,531	-71,375	-89,219
40%	-9,855	-19,710	-29,564	-39,419	-49,274
50%	-1,866	-3,732	-5,597	-7,463	-9,329

Combined, Difference ($, Scenario 2 - 1)	SC Market Share (%)				
BS Market Share (%)	10%	20%	30%	40%	50%
10%	-72,967	-145,934	-218,900	-291,867	-364,834
20%	-61,467	-122,934	-184,400	-245,867	-307,334
30%	-49,967	-99,934	-149,901	-199,867	-249,834
40%	-38,467	-76,934	-115,401	-153,867	-192,334
50%	-26,967	-53,934	-80,901	-107,868	-134,834

Figure 3 **Panel A - Costs**

All Patients	Rapid Infusion (%)	Scenario 1 ($)	Scenario 2 ($)	Difference ($, Scenario 2 - 1)
Payer Costs	5%	5,803,000	5,650,000	-153,000
Payer Costs	50%	5,790,000	5,639,000	-151,000
Payer Costs	100%	5,775,000	5,627,000	-148,000
Provider Costs	5%	5,248,000	5,124,000	-124,000
Provider Costs	50%	5,239,000	5,117,000	-122,000
Provider Costs	100%	5,229,000	5,109,000	-120,000

Figure 3 **Panel B - Hours**

All Patients	Rapid Infusion (%)	Scenario 1 (Hours)	Scenario 2 (Hours)	Difference (Hours, Scenario 2 - 1)
Patient Time	5%	3,110	2,650	-460
Patient Time	50%	2,760	2,360	-400
Patient Time	100%	2,360	2,040	-320
Staff Time	5%	2,300	1,980	-320
Staff Time	50%	2,060	1,790	-270
Staff Time	100%	1,810	1,590	-220

Figure 4 **Panel A - DLBCL+FL**

Percent of patients transitioned to SC (%)	Patient Time (minutes)	Staff Time (minutes)
10%	17	11
20%	35	24
50%	86	58

Figure 4 **Panel B - CLL**

Percent of patients transitioned to SC (%)	Patient Time (minutes)	Staff Time (minutes)
10%	21	14
20%	42	28
50%	106	71

Figure 5 **Panel A - DLBCL+FL - Payer Perspective**

DLBCL & FL, Difference ($, Scenario 2 - 1)	SC Market Share (%)				
BS Market Share (%)	10%	20%	30%	40%	50%
10%	-75	-150	-225	-300	-375
20%	-60	-121	-181	-241	-302
30%	-46	-91	-137	-183	-228
40%	-31	-62	-93	-124	-155
50%	-16	-33	-49	-65	-82

Figure 5 **Panel B - CLL - Payer Perspective**

DLBCL & FL, Difference ($, Scenario 2 - 1)	SC Market Share (%)				
BS Market Share (%)	10%	20%	30%	40%	50%
10%	-245	-491	-736	-981	-1,226
20%	-225	-449	-674	-899	-1,123
30%	-204	-408	-612	-816	-1,021
40%	-184	-367	-551	-734	-918
50%	-163	-326	-489	-652	-815

Figure 5 **Panel A - DLBCL+FL - Provider Perspective**

DLBCL & FL, Difference ($, Scenario 2 - 1)	SC Market Share (%)				
BS Market Share (%)	10%	20%	30%	40%	50%
10%	-58	-115	-173	-231	-289
20%	-44	-88	-132	-176	-220
30%	-30	-61	-91	-122	-152
40%	-17	-34	-50	-67	-84
50%	-3	-6	-10	-13	-16

Figure 5 **Panel B - CLL - Provider Perspective**

DLBCL & FL, Difference ($, Scenario 2 - 1)	SC Market Share (%)				
BS Market Share (%)	10%	20%	30%	40%	50%
10%	-213	-426	-639	-853	-1,066
20%	-194	-388	-582	-776	-970
30%	-175	-350	-525	-700	-875
40%	-156	-312	-467	-623	-779
50%	-137	-273	-410	-547	-683

Figure 6 **Panel A - DLBCL+FL - Costs**

All Patients	Rapid Infusion (%)	Scenario 1 ($)	Scenario 2 ($)	Difference ($, Scenario 2 - 1)
Payer Costs	5%	6,860	6,739	-121
Payer Costs	50%	6,838	6,720	-118
Payer Costs	100%	6,812	6,699	-113
Provider Costs	5%	6,194	6,106	-89
Provider Costs	50%	6,179	6,094	-85
Provider Costs	100%	6,162	6,080	-82

Figure 6 **Panel B - DLBCL+FL - Minutes**

All Patients	Rapid Infusion (%)	Scenario 1 (Minutes)	Scenario 2 (Minutes)	Difference (Minutes, Scenario 2 - 1)
Patient Time	5%	233	199	-35
Patient Time	50%	197	170	-27
Patient Time	100%	156	137	-18
Staff Time	5%	173	149	-24
Staff Time	50%	149	130	-19
Staff Time	100%	123	110	-13
